# Nanoplatelets modified with RVG for targeted delivery of miR-375 and temozolomide to enhance gliomas therapy

**DOI:** 10.1186/s12951-024-02895-6

**Published:** 2024-10-15

**Authors:** Tingting Yang, Nan Zhang, Yuanyuan Liu, Ruyue Yang, Zhaoyi Wei, Futai Liu, Dan Song, Longwei Wang, Jiangyan Wei, Yuanpei Li, Deliang Shen, Gaofeng Liang

**Affiliations:** 1 School of Basic Medicine and Forensic Medicine, Henan University of Science & Technology, Luoyang, 471023 China; 2 Institute of Biomedical Sciences, Henan Academy of Sciences, Zhengzhou, 450009 China; 3 Zhumadian Cental Hospital, Zhumadian, 463000 China; 4 Key Laboratory of Cardiac Injury and Repair of Henan Province, The First Affiliated Hospital of Zhengzhou University, Zhengzhou, 450000 China

**Keywords:** Nanoplatelets, Targeted drug delivery, TMZ, MiR-375, Gliomas

## Abstract

Gliomas are one of the most frequent primary brain tumors and pose a serious threat to people’s lives and health. Platelets, a crucial component of blood, have been applied as drug delivery carriers for disease diagnosis and treatment. In this study, we designed engineered nanoplatelets for targeted delivery of therapeutic miR-375 and temozolomide (TMZ, a first-line glioma treatment agent) to enhance glioma therapy. Nanoplatelets were prepared through mild ultrasound, TMZ and miR-375 were co-loaded through ultrasound and electrostatic interactions, respectively, to combine chemotherapy with gene therapy against glioma. To improve the blood brain barrier (BBB) crossing efficiency and glioma targeting ability, the nanoplatelets were modified with central nervous system-specific rabies viral glycoprotein peptide (RVG) through thiol-maleimide click reaction. The RVG modified nanoplatelets co-loaded TMZ and miR-375 (NR/TMZ/miR-375) not only inherited the good stability and remarkable biocompatibility of platelets, but also promoted the cellular uptake and penetration of glioma tissues, and effectively induced cell apoptosis to enhance the therapeutic effect of drugs. In vivo studies showed that NR/TMZ/miR-375 significantly increased the circulation time of TMZ, and exhibited superior combined antitumor effects. In summary, this multifunctional ‘natural’ nanodrug delivery system provides a potent, scalable, and safety approach for platelet-based combined cancer chemotherapy and gene therapy.

**Supplementary Information:**

The online version contains supplementary material available at 10.1186/s12951-024-02895-6.

## Introduction

Glioblastomas (GBM) are aggressive and lethal tumors that originate from glial cells, which often invade multiple lobes and deep structures of the brain and even affect the contralateral hemisphere [1, 2]. The invasiveness, high genetic heterogeneity, difficulty crossing the blood-brain barrier (BBB), and complex microenvironment of GBM are major challenges in tumor treatment [3, 4]. The current treatment for GBM mainly involves surgical resection, radiotherapy, and chemotherapy with temozolomide (TMZ) [5, 6]. TMZ is crucial in the standard treatment of GBM as a first-line chemotherapy drug. It methylates the guanine and adenine bases of DNA, causing cell DNA double-strand breaks, cycle arrest, and death of tumor cells [7]. However, its further application is limited by the lack of targeting, in vivo instability, and significant hematological toxicity [8]. To improve the therapeutic effect of TMZ, strategies to prolong the half-life, reduce side effects, and target delivery to tumors are urgently needed.

The dysregulation of certain microRNA(miRNA) expression in cells is strongly related to the development and progression of tumors [9]. Blocking carcinogenic miRNAs or introducing tumor-inhibiting miRNAs to reposition miRNA levels in cells to change the pathological status of cells has made effective progress [10, 11]. Relevant studies have shown that miR-375 could inhibit cell proliferation, and cell cycle progression, induce cell apoptosis, and possess the antitumor effect against GBM [12]. And its expression level decreases with the increase of malignancy, which is positively correlated with the prognosis of patients. Overexpression of miR-375 could enhance the therapeutic effect of chemotherapy, thus inhibiting tumor progression [13]. The major challenge in the application of miRNA in vivo is how to deliver it safely and efficiently to tumor cells to increase the expression level, since it is easily degraded in vivo. Nano delivery platforms could reach tumor sites by enhancing permeability and retention (EPR) or active targeting, improving drug utilization, and reducing off-target toxicity, which have attracted wide attention and applied to enhance the therapeutic efficiency of miRNAs [14–17].

Platelets are cytoplasmic fragments produced by the lysis of mature megakaryocytes, with a particle size of 2 ~ 4 μm and a life span of 8 ~ 10d [18], and low immunogenicity. The abundant proteins on the surface of platelets such as CD47, Integrin, CLEC-2 (C-type lectin-like receptor-2), and P-selectin make it superior in immune escape characteristics, crossing BBB and tumor active targeting characteristics [19, 20]. Moreover, the feasibility and cost effective of large manufacturing of nanoplatelets are better than those of nanocarriers such as exosomes, facilitating further application. The above properties, large specific surface area, and good biosafety make platelet-derived nanocarriers an ideal drug delivery platform [21, 22]. Xu et al. prepared platelets that deliver boron nitride (BN-PG) nanoparticles loaded with doxorubicin (DOX) and chlorin e6 for targeted and photodynamic therapy of glioma [23]. Jiang et al. prepared a platelet-based hybrid delivery system (FeDN@P), which integrated dual photothermal, reactive oxygen species-responsive therapeutic nanoparticles and magnetic nanoparticles into platelets to achieve precise photothermal chemotherapy [24]. However, the difficulty of crossing the BBB and the lack of tumor targeting make it difficult to achieve the desired therapeutic effect.

In the current work, we designed a therapeutic targeted nanoplatelet platform for targeted delivery of TMZ and miR-375 to enhance its ability to cross BBB, accumulate in GBM, reduce systemic toxicity, and achieve the ideal combination of gene therapy and chemotherapy. Firstly, washed platelets were prepared through density gradient centrifugation, then polyethyleneimine (PEIs) was modified on the surface of nanoplatelets through electrostatic adsorption to endow nanoplatelets with miR-375 loading capacity. Besides, to enhance the BBB penetration and glioma targeting of nanoplatelets, central nervous system-specific rabies viral glycoprotein (RVG) peptides were modified on the surface of nanoplatelets by click reaction to target nicotinic acetylcholine receptors on the surface of glioma cells [25]. The first-line chemotherapy drug TMZ and therapeutic miR-375 were encapsulated in the nanoplatelets by ultrasonic action and electrostatic action, respectively, to exert combination therapeutic effects.

**Fig. 1:**
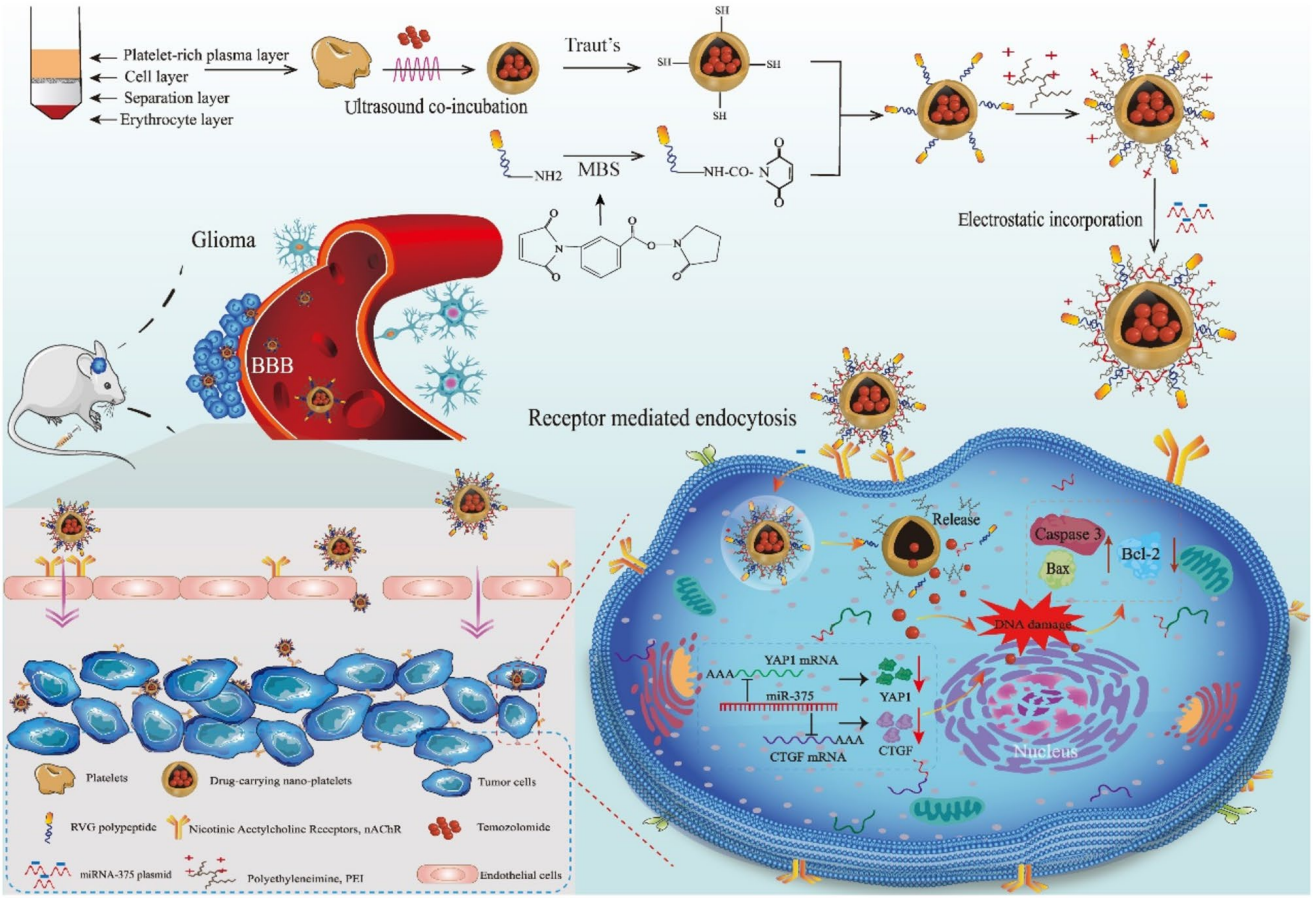
Schematic illustration of synthetic procedure of engineered nanoplatelets and gene therapy combined with chemotherapy against glioma

Herein, the NR/TMZ/miR-375 to enhance glioma therapy were synthesized. NR/TMZ/miR-375 possesses prolonged circulation time in vivo, immune evasion, BBB penetration, and glioma targeting capabilities, exerting potent anti-tumor potency while reducing systemic toxicity. After intravenous injection, nanoplatelets cross the BBB and reach the glioma site due to the effect of RVG, then release TMZ and miR-375 to exert the combined therapeutic effect of chemotherapy and gene therapy, thereby enhancing the antitumor treatment. We evaluated the therapeutic effect of NR/TMZ/miR-375 in an orthotopic mouse glioma model and found that NR/TMZ/miR-375 significantly inhibited tumor proliferation and improved survival rate with good biocompatibility. Altogether, our nanoplatelets can efficiently cross the BBB and actively target tumors with high biosafety, and prolonged circulation time of drug in vivo, which integrates the advantages of chemotherapy and gene therapy, providing a highly valuable therapeutic strategy for the therapy of GBM (See Figure 1).

## Materials and methods

### Materials, cell cultures and animals

The peripheral blood platelet separation solution kits were purchased from Beijing Solaibao Co., Ltd.; RVG-29 peptide was ordered from Nanjing Peptide Biotechnology; DAPI nuclear dye, 1,1′-dioctadecyl-3,3,3′,3′-tetramethy-lindocarbocyanine perchlorate (DiI) fluorescent dye, bicinchoninic acid (BCA) protein assay kit, double antibody (penicillin and streptomycin), PD-10 purification column and cell apoptosis detection kit were purchased from Shanghai Shenggong Co., Ltd.; Thiazole blue tetrazolium bromide (MTT), 1,1’-Dioctadecyl-3,3,3’,3’-Tetramethylindotricarbocyanine Iodide (DiR) fluorescent dye, 2-Iminothiolane hydrochloride (Traut’s), temozolomide (TMZ), and 3-maleimide benzoic acid (MBS) were purchased from Shanghai Butyl Reagent Co., Ltd.; MiRNA-375 plasmid ordered from Genepharma Bio Co., Ltd. Trizol and reverse transcription system were purchased from Beijing Kangwei Century Biotechnology Co., Ltd.; Trypsin and fetal bovine serum were ordered from Vicente Canada Co., Ltd; 3,3′-dioctadecyloxacarbocyanine perchlorate (DiO) fluorescent dye was purchased from Shanghai Biyuntian Co., Ltd.; Fluorescein 5-isothiocyanate (FITC) was purchased from Nanjing Dulai Biotechnology Co., Ltd.

U87 cells, SH-SY5Y cells, and Luciferase U87 (Luc-U87) cells were cultured in DMEM with 10% fetal bovine serum. Balb/c nude mice, 4–6 weeks old, provided by Nanjing Medical Animal Laboratory Center. All animal-related experiments are conducted in accordance with the guidelines for evaluation and approval by the Ethics Committee of Henan Academy of Sciences (HNAS. No20231113b006).

### Preparation of platelets and nanoplatelets

Platelets were isolated by a peripheral blood isolation kit. Briefly, fresh anticoagulant whole blood (heparin anticoagulant) was collected and diluted with whole blood tissue diluent of equal volume. Add at least 5 mL of the separation solution to the 15mL centrifuge tube, the blood was carefully placed on top of the separation solution to maintain a clear boundary between the two solutions. The solution was centrifuged (1200 rpm, 10 min, room temperature, RT) and the supernatant was collected. Then 2 mL PBS was added to the collected solution and centrifuged (4000 rpm, 20 min, RT), and the supernatant was removed. 3 mL PBS was used to re-suspend the obtained platelets, and the precipitate was collected after centrifugation (4000 rpm, 20 min, RT). Finally, the platelets were re-suspended with PBS buffer containing low concentration aspirin and stored at 4 ℃ for further studies.

The platelets after fresh washing were treated under different ultrasonic conditions with the probe ultrasonic device. The size of the nanoparticles was analyzed by the Zetasizer-Nano-ZS system to obtain the optimal ultrasonic conditions. The obtained bare nanoplatelets (NP) were filtered by a 0.22 μm filter, and the protein concentration was detected by the BCA method and stored at 4 ℃.

The RVG polypeptide was modified onto the surface of NP (NR) by thiol-maleimide click reaction and evaluated by fluorescence colocalization. RVG and FITC were dissolved with PBS and DMSO at concentrations of 1 mg/mL and 0.5 mg/mL, respectively. The RVG peptide (1 mg/mL, 40 µL) was incubated with FITC (0.5 mg/mL, 1 µL) for 30 min (37 ℃) to obtain FITC-labeled RVG (FITC-RVG). Then FITC-RVG was incubated with MBS (0.1 mg/mL, 1 µL) for 30 min to generate maleimide groups, the free MBS was removed by centrifugal filtration (MWCO 8000 Da).

DiI, a red membrane dye, was dissolved in DMSO (0.5 mg/mL) to label the nanoplatelets (DiI-NP). Briefly, DiI (0.5 mg/mL, 1 µL) was incubated with nanoplatelets (1 mg/mL, 200 µL) for 30 min at 37 ℃. Then the DiI-labeled nanoplatelets (DiI-NP) were incubated with 0.1 mg/mL of Traut’s reagent (1 µL) for 30 min (37 ℃) to generate sulfhydryl groups on the surface of the nanoplatelets. Finally, FITC-RVG was incubated with DiI-NP for 1 h (37 ℃) to obtain RVG modified NPs and assessed using confocal laser scanning microscopy (CLSM). The procedure for RVG modification of nanoplatelets was similar to that described above.

### Preparation and characterization of drug-loaded nanoplatelets

TMZ was dissolved in DMSO (200 µM) and stored at -20℃. To explore the optimum drug loading efficiency. Nanoplatelets (1 mL, 2 mg/mL) were mixed with serial concentrations of TMZ (0.1 mL) at room temperature for 30 min, and the mass ratio (nanoplatelets: TMZ) was 1:2.5, 1:5, 1:10, 1:20 and 1:40, respectively. The mixture was then sonicated at 200 W and 120 cycles (1 cycle: sonication for 5s, pause for 5s) and finally placed in a cell incubator for 1 h to recover the cell membrane. Subsequently, free TMZ was removed through by dialysis in deionized water, and the concentration of TMZ was detected by UV-Vis spectrophotometer at 328 nm. To calculate the drug loading efficiency (DLE) and encapsulation efficiency (EE), to determine the optimal conditions for ultrasonic co-incubation. The formula for calculating DLE and EE was as follows:

$$\:DLE\left(\%\right)=\frac{Mass\:of\:total\:drugs\:in\:system}{Total\:mass\:of\:system}\times\:100\%$$

$$\:EE\left(\%\right)=\frac{Total\:drug-Free\:drug}{Total\:drug}\times\:100\%$$

PEI was applied as a drug carrier to endow nanoplatelet miRNA loading capacity. 10 mg PEI was dissolved in deionized water (10 mL), and stored at 4 ℃. Then 10 µL PEI (1 mg/mL) was incubated with 1 mL of various concentrations of nanoplatelets for 30 min (The mass ratio of PEI to nanoplatelets: 1:0, 1:0.2, 1:0.5, 1:1, 1:2, 1:3, 1:4, 1:5, 1:6). Then the zeta potential of PEI modified nanoplatelets were studied to detect the suitable modification conditions of PEI.

To prepare the miR-375 loaded nanoplatelets, the PEI-modified NR/TMZ (1 mg/mL, 0.5mL) were mixed with miR-375 plasmid in different proportions (The mass ratios of NR/TMZ to miR-375 plasmid: 10:1, 10:2, 10:3, and 10:4). Subsequently, the nanoplatelets loaded with miR-375 plasmids were evaluated by electrophoresis (80 V, 50 min) on 1% agarose gel.

Transmission electron microscope (TEM) and DLS characterization of drug-loaded nanoplatelets according to the previous works [18].

### In vitro stability and drug release of NR/TMZ/miR-375

Bare nanoplatelets (NP) and NR/TMZ/miR-375 (0.1 mL, nanoplatelets: 1 mg/mL) were dispersed in PBS buffer with pH 7.4. The particle size changes of NP and NR/TMZ/miR-375 were observed within 7 days by the Marvin size analyzer.

The in vitro drug release was evaluated through the dialysis method. NP loaded with TMZ (NP/TMZ, 0.5 mL, 1 mg/mL) and NR/TMZ/miR-375 (0.5 mL, 1 mg/mL) were dispersed in 2 mL PBS buffer solution with pH 7.4. The solution was then put into a dialysis bag (MWCO 3500 Da), immersed in glass jars containing PBS with pH 7.4, and shaken at 37 ℃ (100 rpm) in a shaker. 0.1 mL of PBS in the glass dish was taken for detection at 0.5, 1, 2, 3, 4, 5, 6, 12 and 36 h. The concentration of TMZ in the solution was detected by UV-Vis spectrophotometer at 328 nm.

### In vitro tumor targeting and glioma organoids penetration detection

U87 cells (5 × 10^5^ cells/mL) were seeded in glass bottom culture dishes, and incubated with 10 µM DiO for 10 min. Similarly, NP, RVG modified nanoplatelets (NR), and NR/TMZ/miR-375 were labeled with DiI (NP concentration:10 µL, 1 mg/mL), and incubated with DiO labeled cells and observed by CLSM.

Glioma organoids were constructed according to the previous studies [26]. When the diameter of the organoids was about 150 μm, the glioma organoids were then separately treated with DiI labeled NP, NR, and NR/TMZ/miR-375 (nanoplatelets concentration: 10 µL, 1 mg/mL) on glass bottom culture dishes and studied with CLSM.

### MTT, cell migration, cell apoptosis assay

The cytotoxicity of engineered nanoplatelets to U87 and SH-SY5Y cells were determined by MTT assay according to our previous study [27]. Cells were seeded in the 96 well plate (5 × 10^3^ cells/well), treated with PBS, NP, bare nanoplatelets loaded with miR-375 (NP/miR-375), RVG modified nanoplatelets loaded with miR-375 (NR/miR-375), RVG modified nanoplatelets loaded with TMZ (NR/TMZ) and NR/TMZ/miR-375, respectively (TMZ: 80 µg/mL, miR-375: 4 µg/mL). After 24 h, the medium was removed and cells were incubated with MTT solution (20 µL, 5 mg/mL) for 4 h. Then MTT solution was replaced with DMSO and the formazan was analyzed through a microplate reader at 490 nm.

The effect of engineered nanoplatelets on the migration ability of U87 cells and SH-SY5Y cells were investigated by the cell wound healing experiment. Cells were seeded in 6 well plates (5 × 10^5^ cells/well) and the wound was made through pipettes. Then cells were treated with NP, NP/miR-375, NR/miR-375, NR/TMZ, and NR/TMZ/miR-375 (TMZ: 80 µg/mL, miR-375: 4 µg/mL) for 0, 24, and 48 h.

Cell apoptosis was measured by flow cytometry. The cells were separately seeded in 6 well plates (5 × 10^5^ cells/well) and treated with NP, NP/miR-375, NR/miR-375, NR/TMZ and NR/TMZ/miR-375 (TMZ: 80 µg/mL, miR-375: 4 µg/mL). After 48 h, an Annexin V-FITC/PI cell apoptosis detection kit was used to stain the cells according to standard instructions.

### Expression of miR-375 gene in U87 and SY5Y cells

U87 and SH-SY5Y cells were seeded in 6 well plates (5 × 10^5^ cells/well) and treated with NP, NP/miR-375, NR/miR-375, NR/TMZ and NR/TMZ/miR-375 for 48 h. After treatment, RNA was extracted with Trizol reagent, and quantitative real-time reverse transcription PCR (qRT-PCR) was performed according to the instructions. The relative expression of miR-375 was calculated according to 2^−ΔΔCT^ methods (See Table 1).

**Table 1:** The sequence of PCR primers used

Name of primer	Forward primer (5’ to 3’)	Reverse primer (5’ to 3’)
RT-miR-375	CTCAACTGGTGTCGTGGAGTCGGCAATTCAGTTGAGTCACGCGA	
miR-375	ACACTCCAGCTGGGTTTGTTCGTTCGGCTC	TGGTGTCGTGGAGTC
RNU6B	GCTTCGGCAGCACAT ATACTAAAAT	CGCTTCACGAATTTGCGTGTCAT

### Western blot analysis

U87 cells were seeded in 6 well plates (5 × 10^5^ cells/well) and separately incubated with PBS, NR/TMZ, NR/miR-375, and NR/TMZ/miR-375 (TMZ: 80 µg/mL, miR-375: 4 µg/mL) for 24 h, and the total cellular protein extraction and protein quantification were performed according to the previous studies [20]. The proteins were treated with 12% sodium dodecyl sulfate-polyacrylamide gels and transferred to a polyvinylidene fluoride (PVDF) membrane, then blocked for 1 h, stained with primary antibodies YES-related protein (YAP1), Connective tissue growth factor (CTGF), cleaved Caspase3, BCL2-associated X protein (Bax), and B cell lymphoma-2 (Bcl-2) for 12 h (4 °C), stained with secondary antibody for 2 h, visualized through ECL detection system (Tanon5200, China).

### In vivo biodistribution of NR/TMZ/miR-375

All the animal experiments were approved by the Ethics Committee of Henan Academy of Sciences. Subcutaneous and orthotopic glioma xenograft models were established using Luc-U87 cells [28–31] for the in vivo biodistribution study. The mice applied for subcutaneous glioma models and orthotopic glioma models were injected with 1 × 10^6^ cells per mouse, and 5 × 10^5^ cells per mouse, respectively. The tumor-bearing Balb/c nude mice were randomly divided into two groups and injected with DiR-labeled NP and DiR-labeled NR/TMZ/miR-375 (DiR: 1 µM) through the tail vein, respectively. After 0 h, 8 h, and 12 h, the distribution of nanoplatelets in mice was observed by the IVIS imaging system (PerkinElmer, Lumina III). Then the main organs were collected and imaged.

### In vivo pharmacokinetic and antitumor efficiency study

Before conducting anti-tumor studies, the pharmacokinetics of TMZ were first analyzed. The mice were randomly divided into 3 groups, which were administrated with saline, TMZ, and NR/TMZ/miR-375 (TMZ: 40 mg/kg) through tail vein respectively. And the blood of mice in different groups were collected through tail vein to detect the TMZ concentration at the same time point.

Orthotopic glioma xenograft models were utilized to analyze the antitumor effect in vivo. Luc-U87 cells (1 × 10^8^ cells/mL, 5 µL per mouse) were applied for the tumor model according to previous studies [30]. Ten days after tumor transplantation into the brain of nude mice, mice with similar fluorescence intensity were randomly divided into 4 groups and administered with saline, NR/miR-375, NR/TMZ, and NR/TMZ/miR-375 (TMZ:40 mg/kg, miR-375: 0.2 mg/kg) through tail vein every 3 days and total 5 times. On the 10th, 13th, 16th, 19th, and 22nd days, mice were intraperitoneally administered with luciferase substrate, and the luminescence of the tumor was studied by an in vivo imaging system (IVIS, PerkinElmer, Lumina III). The mice were weighed every 3 days. After the treatment, the mice were anesthetized and sacrificed to obtain major organ tissues for H&E assay.

### Statistical analysis

Data were expressed as mean and standard deviation (mean ± SD). All statistical tests were performed in SPSS Statistics 23, and one-way analysis of variance (ANOVA) test was used for statistical analysis; *P* < 0.05 (*) and *P* < 0.01 (**) were considered statistically significant.

## Results and discussion

### Preparation and characterization of NR/TMZ/miR-375 nanoplatelets

**Fig. 2:**
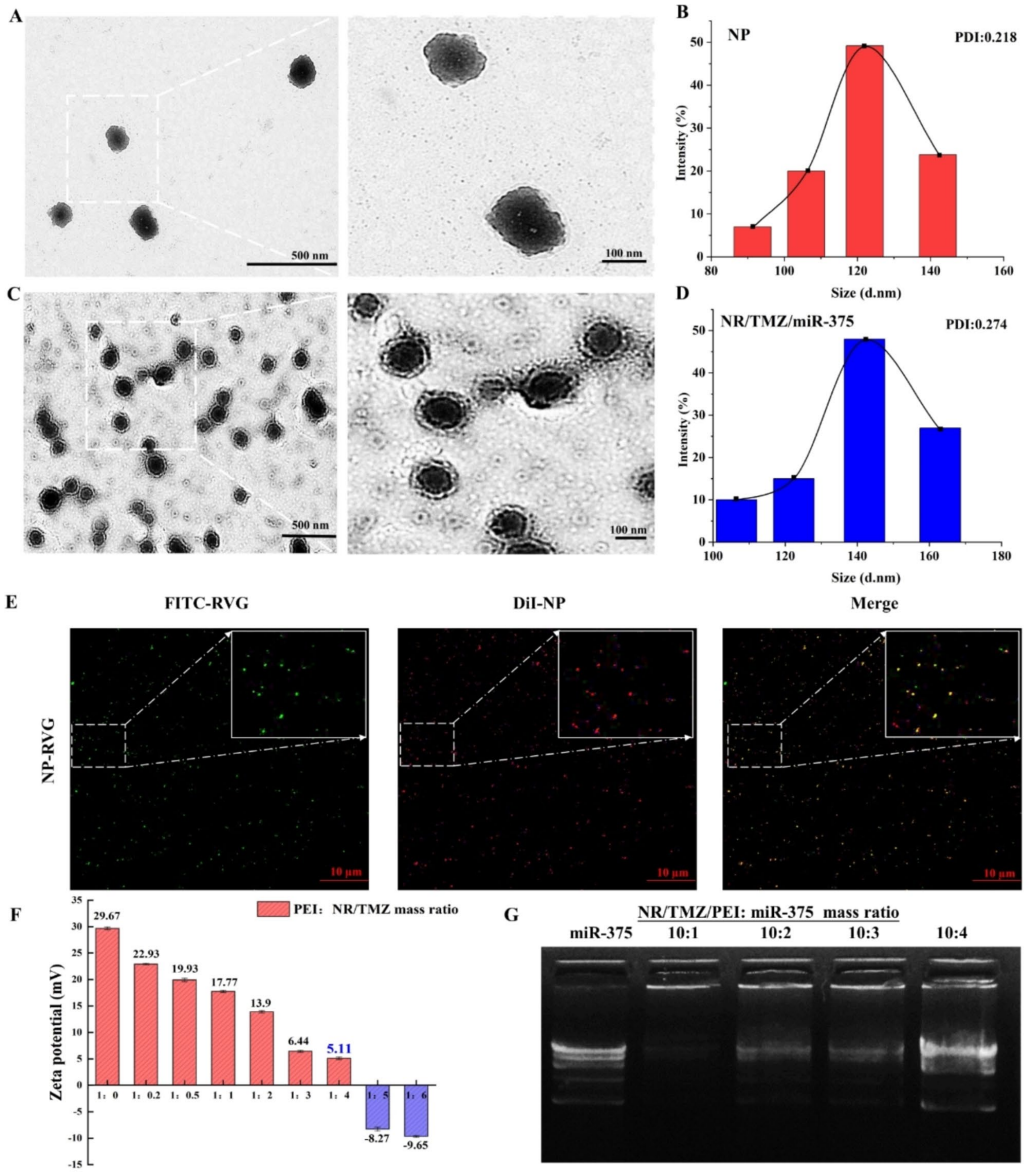
Characterization of nanoplatelets. (**A**) TEM images of NPs. Scale bar: 500 nm (left), 100 nm (right). (**B**) DLS particle size distribution of NPs. (**C**) TEM images of NR/TMZ/miR-375. Scale bar: 500 nm (left), 100 nm (right) (**D**) DLS particle size distribution of NR/TMZ/miR-375. (**E**) CLSM images of nanoplatelets (DiI) modified with RVG (FITC). (**F**) Zeta potential analysis of nanoplatelets modified with various concentrations of PEI. The results are shown as mean ± standard deviation, *n* = 3. (**G**) Results of agarose gel electrophoresis analysis of nanoplatelets incubated with various concentrations of miR-375

**Fig. 3:**
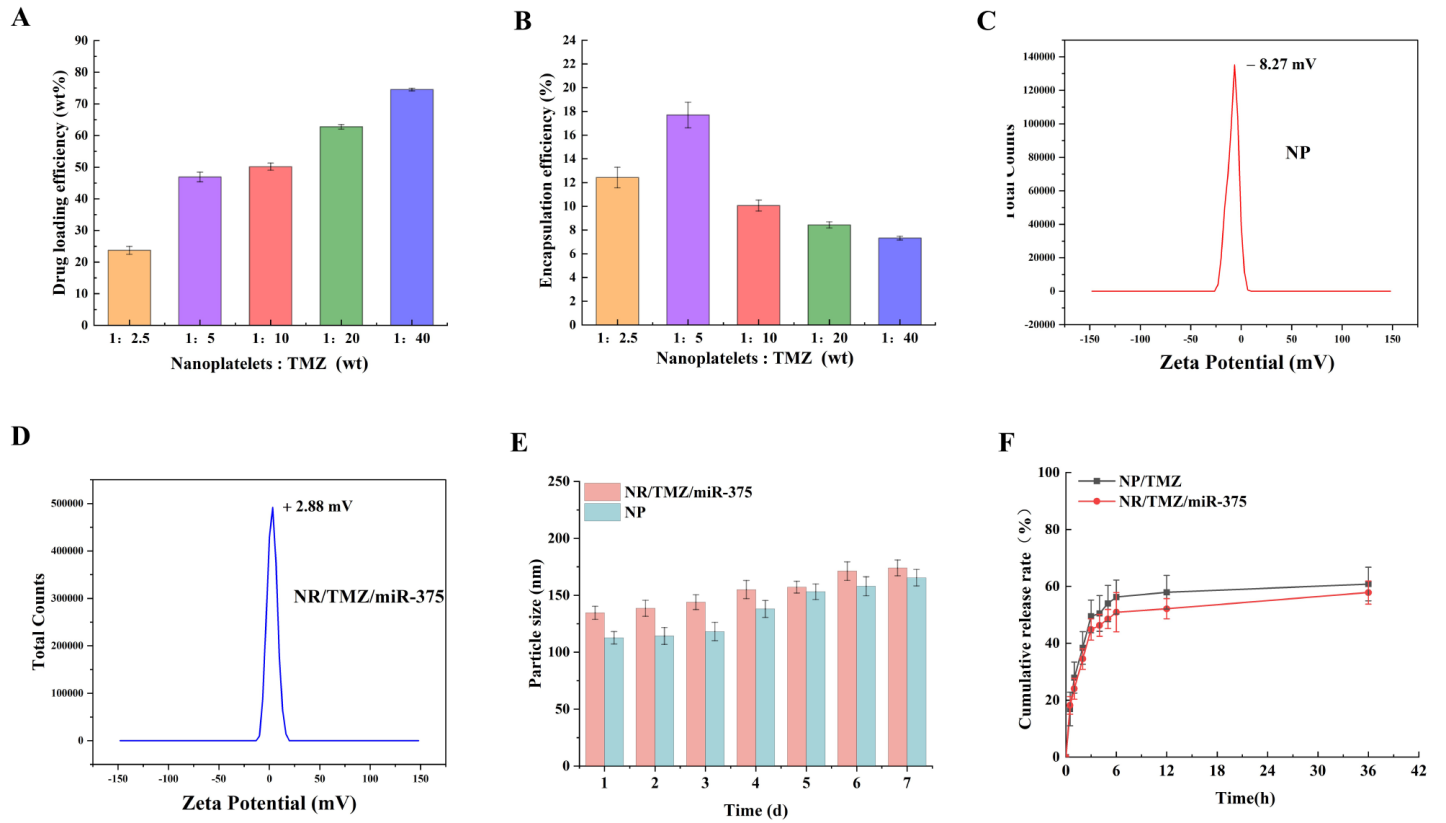
Drug loading properties and stability of nanoplatelets. (**A**) DLE and (**B**)EE of TMZ in nanoplatelets incubated with different concentrations of TMZ. (**C**) Zeta potential of NP and (**D**) NR/TMZ/miR-375. (**E**) Stability study of NP and NR/TMZ/miR-375 in PBS (pH 7.4, *n* = 3) within 7 days. (**F**) Kinetics of cumulative release of TMZ from NP/TMZ and NR/TMZ/miR-375 in PBS (pH 7.4, *n* = 3)

Platelets were prepared from whole blood by gradient centrifugation, and the nanoplatelets obtained under different ultrasonic conditions were shown in Tab. S1. Among them, ideal nanoplatelets could be obtained under the conditions of 300 W and 120 cycles (Ultrasound 5s, pause 10s, for a cycle). The morphology of bare nanoplatelets (NP) were observed by TEM. As shown in Fig. 2A and B, NPs were uniformly spherical with a mean size of 123 ± 9 nm. Then RVG polypeptides were coupled with NPs through a thiol-maleimide click reaction, and examined by CLSM. The result (Fig. 2E) showed that the green fluorescence (RVG-FITC) and red fluorescence (DiI-NPs) overlapped into yellow (RVG-NPs), suggesting RVG polypeptides were successfully conjugated to nanoplatelets. Different concentrations of TMZ were encapsulated into nanoplatelets by ultrasound to study the optimal DLE. The results were shown in Fig. 3A and B, DLE gradually increased with the increase of TMZ concentration, but EE showed a trend of first increasing and then decreasing. Since the mass of loaded drug remained unchanged with the increasing of feeding amount, 1:20 (mass ratio of NP: TMZ) was chosen for further study (DLE: 62.64 ± 0.72%, EE: 8.43 ± 0.26%). PEI was applied to endow nanoplatelets with miR-375 encapsulation capability. Since the surface charge of nanoparticles played an important role in cellular internalization, it had been shown that nanoparticles with micro-positive charges could not only escape the “protein crown” effect, but were also readily adsorbed by anionic cell membranes, enhancing cellular uptake [32, 33]. PEI was incubated with a series of NR/TMZ, mass ratio of PEI to NR/TMZ was 1:0, 1:0.2, 1:0.5 1:1, 1:2, 1:3, 1:4, 1:5, 1:6. The formulations were continuously optimized using a zeta potential analyzer. Figure 2F showed that when the mass ratio was 1:4, the zeta potential of the optimal TMZ-loaded RVG and PEI-modified nanoplatelets (NR/TMZ/PEI) reached 5.11 ± 0.18 mV, which was a micro-positive potential and selected for miR-375 loading. Serial concentrations of miR-375 plasmid were incubated with NR/TMZ/PEI, and evaluated through gel retardation assay. According to the results in Fig. 2G, when the mass ratio of NR/TMZ/PEI: miR-375 was 10:1, the plasmid was fully loaded (DLE: 9.1%), which was utilized for subsequent studies.

TEM images of NR/TMZ/miR-375 were shown in Fig. 2C, NR/TMZ/miR-375 was observed to be spherical with an obvious membrane-like structure, and its mean size was about 144 ± 14 nm, which was slightly larger than NPs, probably due to the modification of surface adsorbed PEI with miR-375 and RVG which increased the particle size. And the zeta potential of NR/TMZ/miR-375 was + 2.88 mV while that of NP was − 8.27 mV (Fig. 3C and D), indicating that NR/TMZ/miR-375 was successfully endowed with micro-electro-positivity by adjusting the formulation of nanoplatelets.

Stability is a vital property of nanoparticles. The stability study result was shown in Fig. 3E, the particle size of NR/TMZ/miR-375 and NPs depicted a slightly increasing trend within 7 days in PBS (pH = 7.4). This may be related to the presence of salt ions on the surface of the particles, which almost did not affect the stability of the nanoparticles and was conducive to further in vivo studies. Then the sustained drug release ability of the NPs was measured by dialysis method. The drug release curve was shown in Fig. 3F. Within 4 h, the cumulative release of TMZ from NR/TMZ/miR-375 and bare nanoplatelets loaded with TMZ (NP/TMZ) in PBS was about 44.84 ± 3.73%, and 49.47 ± 5.75%, respectively, indicating the good drug sustained release ability of NR/TMZ/miR-375, and the modification of nanoplatelets did not affect its drug release property.

### In vitro cellular uptake and penetration evaluation

**Fig. 4:**
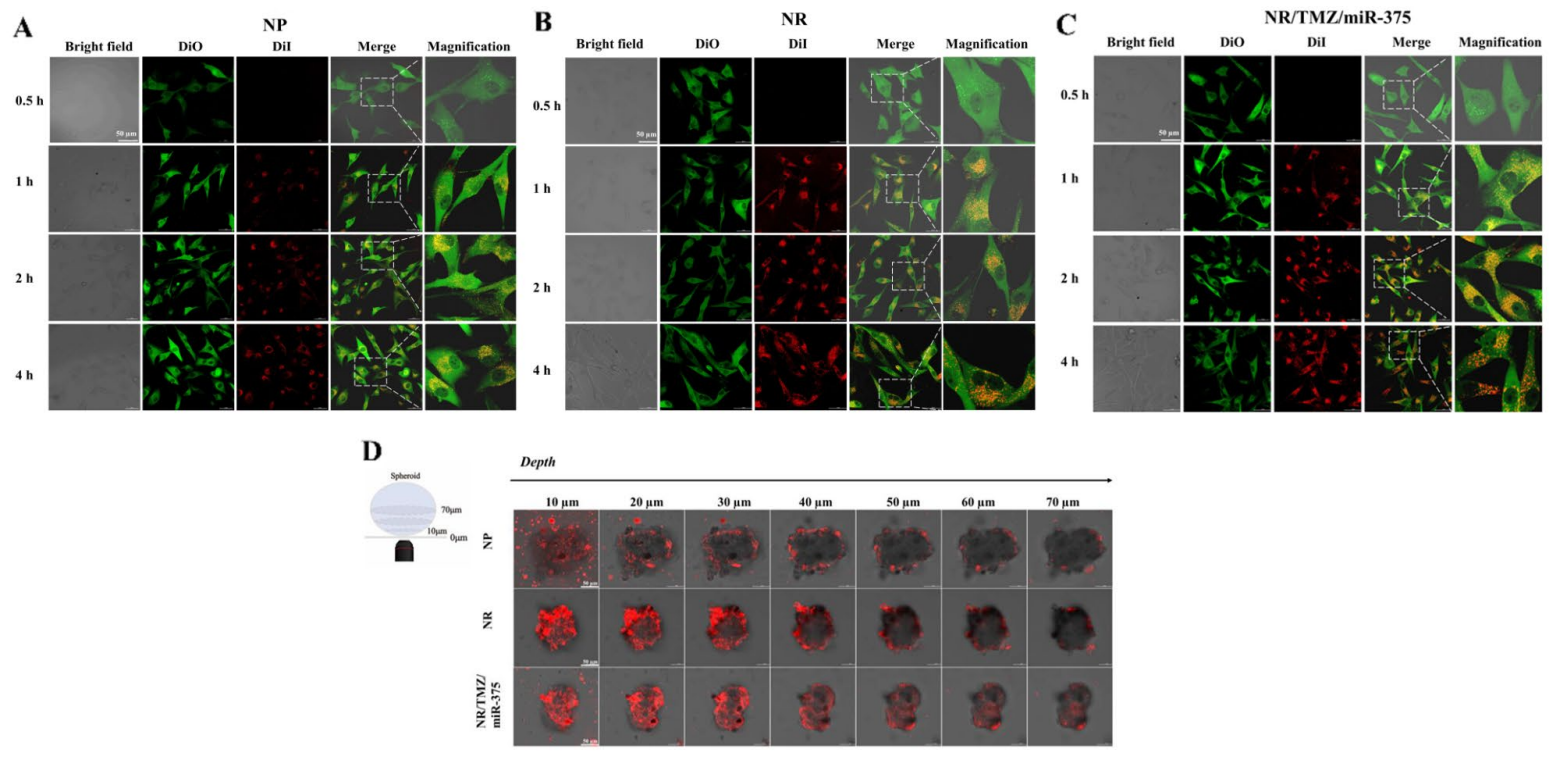
In vitro cellular uptake and penetration evaluation of nanoplatelets. The cellular uptake of (**A**) NP, (**B**) NR and (**C**) NR/TMZ/miR-375 by U87 cells at different time points was observed by CLSM (scale bars 50 μm). (**D**) Schematic illustrates of in vitro penetration evaluation (left), distribution of NP, NR and NR/TMZ/miR-375 in glioma organoids (scale bars 50 μm)

**Fig. 5:**
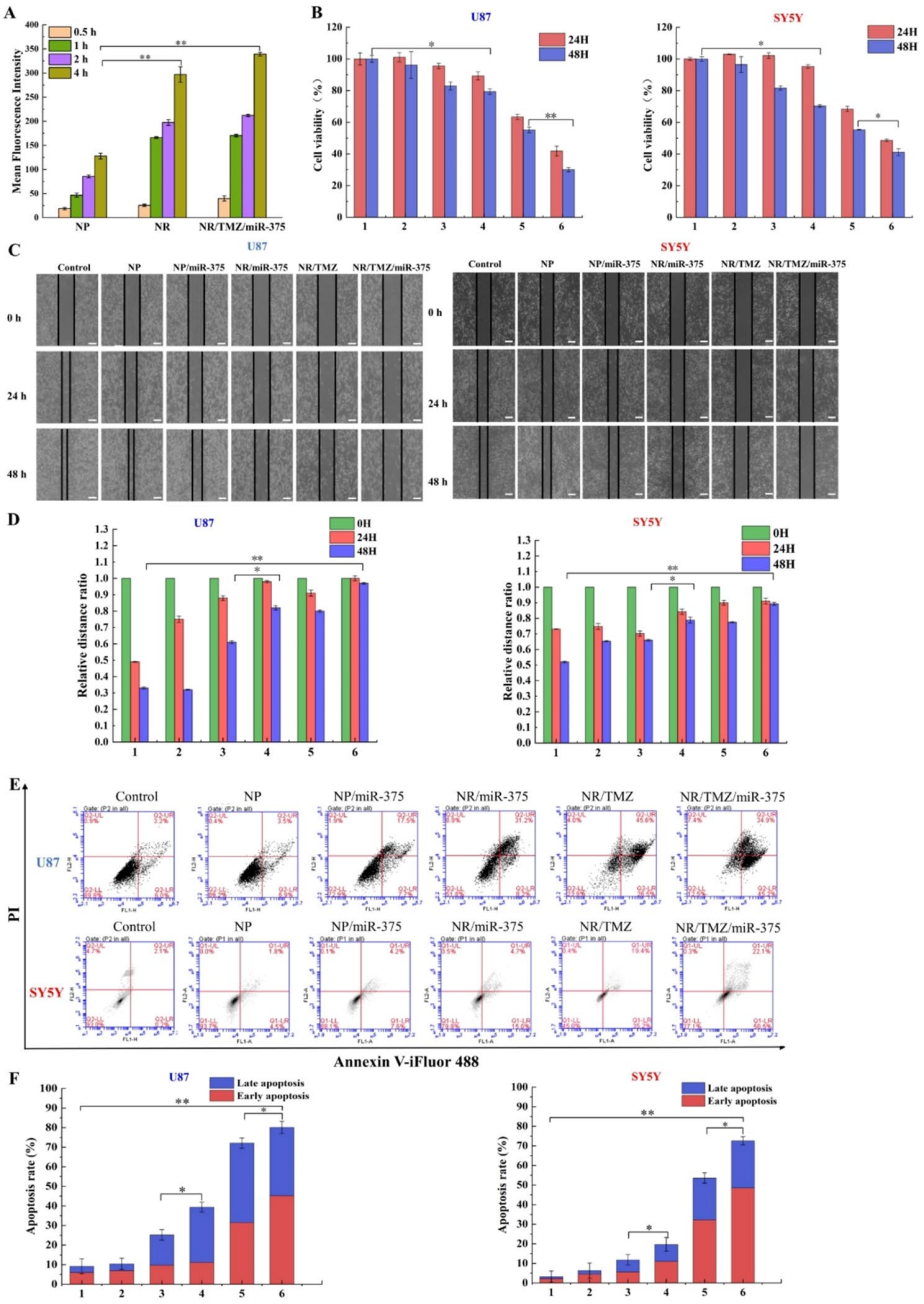
(**A**) Quantitative statistical results of cellular uptake. (**B**) The effects of different nanoparticles on the cell viability of U87 and SH-SY5Y cells for 24 h and 48 h. (**C**) The effect of engineered nanoplatelets on the migration of U87 and SY5Y cells and (**D**) the statistical results of the relative migration distance ratio; (**E**) Flow cytometry results of U87 and SY5Y cells treated with different groups and (**F**) statistical results of apoptotic rate. (1: control, 2: NP, 3: NP/miR-375, 4: NR/miR-375, 5: NR/TMZ, 6: NR/TMZ/miR-375; The results are shown as mean ± standard deviation, *n* = 3, *P < 0.05, **P < 0.01 was statistically significant)

RVG is a polypeptide derived from the neurotropic domain of rabies virus glycoprotein, which controls three acetylcholine receptors and assists in the non-invasive delivery of particles along the motor neuron retrograde into the central nervous system [34]. To investigate the targeting efficiency of RVG peptide-modified nanoplatelets, NP, NR and NR/TMZ/miR-375 were incubated with U87 cells, then samples and U87 cell membranes were labeled with DiI and DiO, respectively, and the results were observed by CLSM (Fig. 4A-C). The fluorescence intensity of DiI (red fluorescent) was positively correlated with the incubation time, indicating that the nanoplatelets were efficiently internalized by U87 cells. After 4 h, the DiI fluorescence intensity (red) of NR and NR/TMZ/miR-375 groups were significantly enhanced than NP, which was 2.32 and 2.65 times higher than NP group (Fig. 5A), respectively, implying that RVG modification greatly improved the cellular uptake of nanoplatelets.

In order to study the penetration ability of engineered nanoparticles, we cultured glioma organoids, as shown in Fig. S2. After 120 h, it was found that most cells could form organoids with a diameter of about 150 μm, which proved that the organoids model was successfully established. We then incubated DiI-labeled NP, NR, and NR/TMZ/miR-375 with organoids and investigated the penetration ability. After 4 h of co-incubation, weak red fluorescence signals in the outer layer could be observed in the NP group (Fig. 4D). At the depth of 50 μm, there was almost no red fluorescent signal of NP group. In contrast, the red fluorescence of NR and NR/TMZ/miR-375 groups were distributed throughout the organoids, especially in the center of the organoids at a depth of 70 μm, and the accumulation of NR and NR/TMZ/miR-375 in the organoids was considerably higher than that in the NP group. The above results prove that RVG modification could promote the cellular uptake and cell penetration of nanoplatelets, which is consistent with the existing previous research, thus exerting an efficient antitumor effect in vivo [34].

### Antitumor effect in vitro

It is verified that RVG could promote the cell penetration of nanoplatelets, we analyzed whether RVG can assist nanoplatelets to exert cytotoxicity, which was the key issue for efficient antitumor therapy. Before studying the cytotoxicity of nanoplatelets. Before analyzing the cytotoxicity of nanoplatelets, the biocompatibility of nanoplatelets was first studied. The biocompatibility of 293T cells treated with nanoplatelets was studied by MTT method. The cell viability was still over 90% at the nanoplatelets concentration of 40 µg/mL. When it increased to 800 µg/mL, cell viability was almost about 90%, demonstrating the superior biosafety of nanoplatelets (Fig. S3). Then we explored the antitumor effect of nanoplatelets on U87 and SH-SY5Y cells (Fig. 5B). In both cell lines, there was no observably inhibitory effect in the NP group, while the cell viability of NR/miR-375 group was evidently lower than that of NP/miR-375, this may be ascribed to the enhanced therapeutic effect of miR-375 by the targeting effect of RVG. Notably, the cell viability of U87 cells in NR/TMZ/miR-375 group was only 30.1% and that of SH-SY5Y cells was 39.5% at 48 h, which was considerably lower than that of the single-loaded groups (NR/TMZ and NR/miR-375), suggesting the superiority of combined therapy.

Wound healing assay was performed to detect effect of engineered nanoplatelets on the migration of U87 cells and SH-SY5Y cells (Fig. 5C, D). The cell migration distance in the NP/miR-375 group was greater than that in the NR/miR-375 group. Compared to the single-loading group, the NR/TMZ/miR-375 group exhibited the greatest relative distance in the two cell lines, indicating a remarkable inhibition effect on cell migration. These results reflected the ability of RVG to enhance the therapeutic effect of drugs and revealed the favorable therapeutic effect of combined therapy.

The enhanced therapeutic effects were further assessed by flow cytometry. Series nanoparticles were treated with U87 and SH-SY5Y cells for 48 h, cell apoptosis rate results were shown in Fig. 5E, F consistent with the results of cytotoxicity, the apoptosis rate of NR/miR-375 group was higher than that of NP/miR-375 group, which was 1.56 times higher than that of NP/miR-375 group after 48 h of nanoplatelets treatment, suggesting that RVG could promote the antitumor effect of drug loaded in nanoplatelet. And the NR/TMZ/miR-375 group showed the highest apoptosis rate (80.1% in U87 cells and 72.6% in SH-SY5Y cells), reflecting the superior therapeutic effect of NR/TMZ/miR-375.

**Fig. 6:**
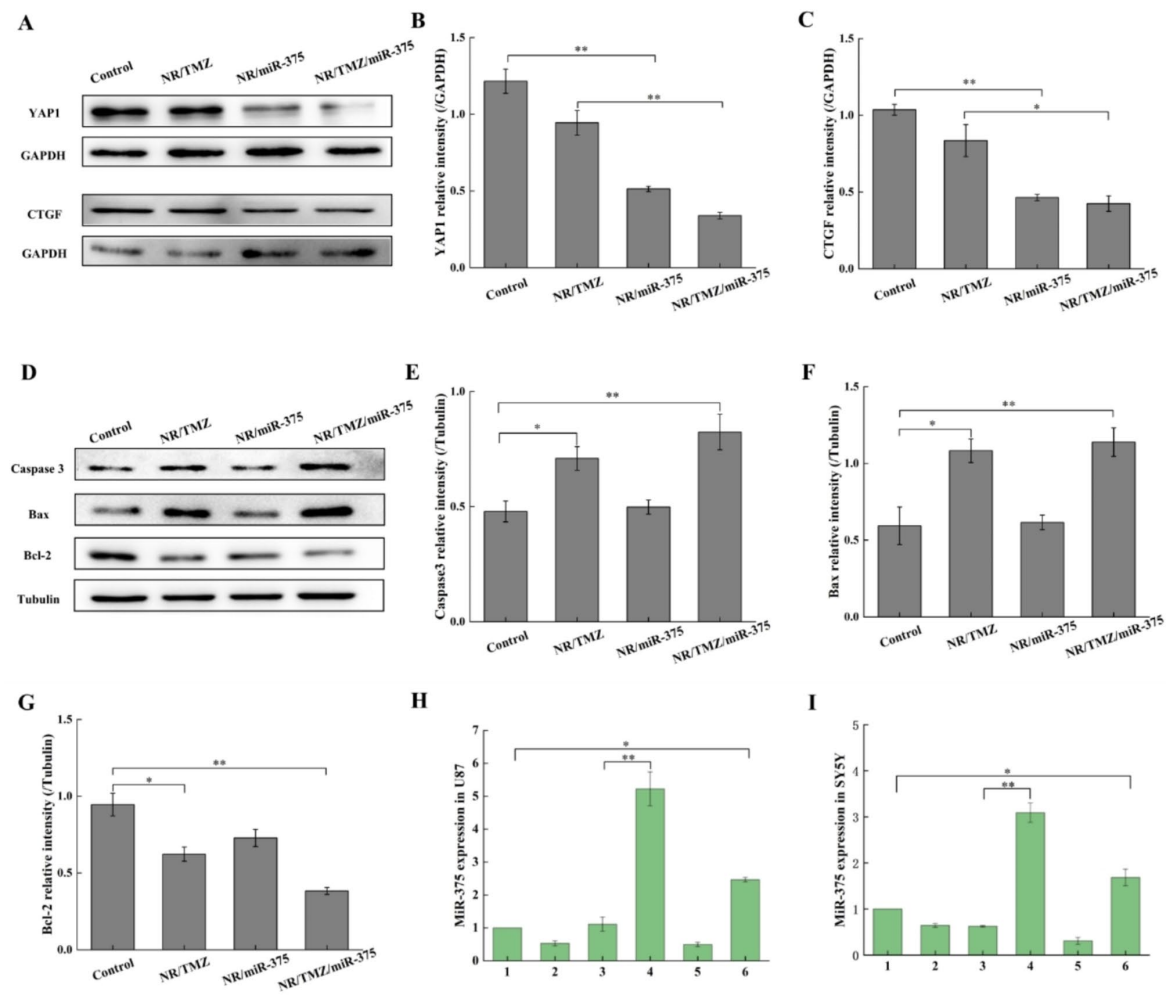
Western blot studies of U87 cells treated with nanoplatelets. (**A**) The expression level of miR-375 target protein was detected by Western blot. (**B**, **C**) Relative quantitative statistical results of YAP1 and CTGF protein expression. (**D**) Expression levels of apoptosis-related proteins in different experimental groups. (**E**-**G**) Relative quantitative statistical results of cleaved Caspase3, Bax, and Bcl-2 protein expression. (**H**) miR-375 gene expression in U87 and SH-SY5Y (**I**) cells. (1: control, 2: NP/NC, 3: NP/miR-375, 4: NR/miR-375, 5: NR/TMZ, 6: NR/TMZ/miR-375; The results are shown as mean ± standard deviation, *n* = 3, *P < 0.05, **P < 0.01 was statistically significant)

Next, we explored the mechanism of combined miR-375 and TMZ affecting glioma cell apoptosis through western blot experiments. YAP1, a YES-related protein, was involved in intracellular signal transduction and transcriptional coactivation of downstream target molecules in the form of phosphorylation, and its expression level was positively correlated with tumor evolution and poor prognosis [35, 36]. Ding et al. found that YAP1 was critical in the pathogenesis of glioma, was overexpressed in glioma cells [37], and was the target gene of miR-375. Connective tissue growth factor (CTGF) is a stromal cell protein that is associated with tumor progression with significantly decreased expression level of the connective tissue compared with glioma tissue [38, 39]. Zhang et al. found that miR-375 can interact with the 3 ‘-UTR of CTGF mRNA, proving that CTGF is one of the direct targets of miR-375 [40]. Results as shown in Fig. 6 (A-G), compared with the control group and NR/TMZ group, the expression levels of YAP1 and CTGF proteins in the NR/miR-375 and NR/TMZ/miR-375 groups were markedly down-regulated. YAP1 and CTGF expression in the NR/TMZ/miR-375 group were 0.27 times and 0.41 times of those in the control group, respectively, suggesting that the nanoplatelets successfully delivered miR-375 to glioma cells, and effectively exerted therapeutic effects. Meanwhile, pro-apoptotic proteins Bax, cleaved Caspase 3 and anti-apoptotic protein Bcl-2 were selected to investigate the effects of NR/TMZ/miR-375 on the expression of apoptosis-related proteins in U87 cells [41]. The expression levels of Bax and Caspase 3 in NR/TMZ group were increased by 1.48 and 1.82 times, respectively, while Bcl-2 was decreased by 34.14% compared with control. In the NR/TMZ/miR-375 group, the expressions of Bax and cleaved Caspase 3 were increased by 1.72-fold and 1.92-fold, respectively, while Bcl-2 was decreased by 59.52% compared with control, suggesting that the co-treatment of the two drugs could significantly enhance apoptosis. Compared with the control group, there was no notable difference in NR/miR-375, indicating that it could not induce the changes of the three proteins under the experimental conditions. Figure 6H and I present the results of real-time fluorescent quantitative PCR, illustrating the expression of miR-375 in tumor cells after 24 h of treatment in various dosing groups. The findings indicate a significant up-regulation of miR-375 in both U87 and SH-SY5Y cells within the NR/miR-375 administration group, which was consistent with the results of the western blot studies. In conclusion, NR/TMZ/miR-375 greatly reduced the expression of miR-375 targeted proteins YAP1 and CTGF, increased expression of apoptosis-related proteins Bax and cleaved-Caspase3, decreased Bcl-2 expression, respectively, successfully activated the Caspase-dependent apoptotic pathway to inhibit the proliferation of U87 cells.

### In vivo targeting study

**Fig. 7:**
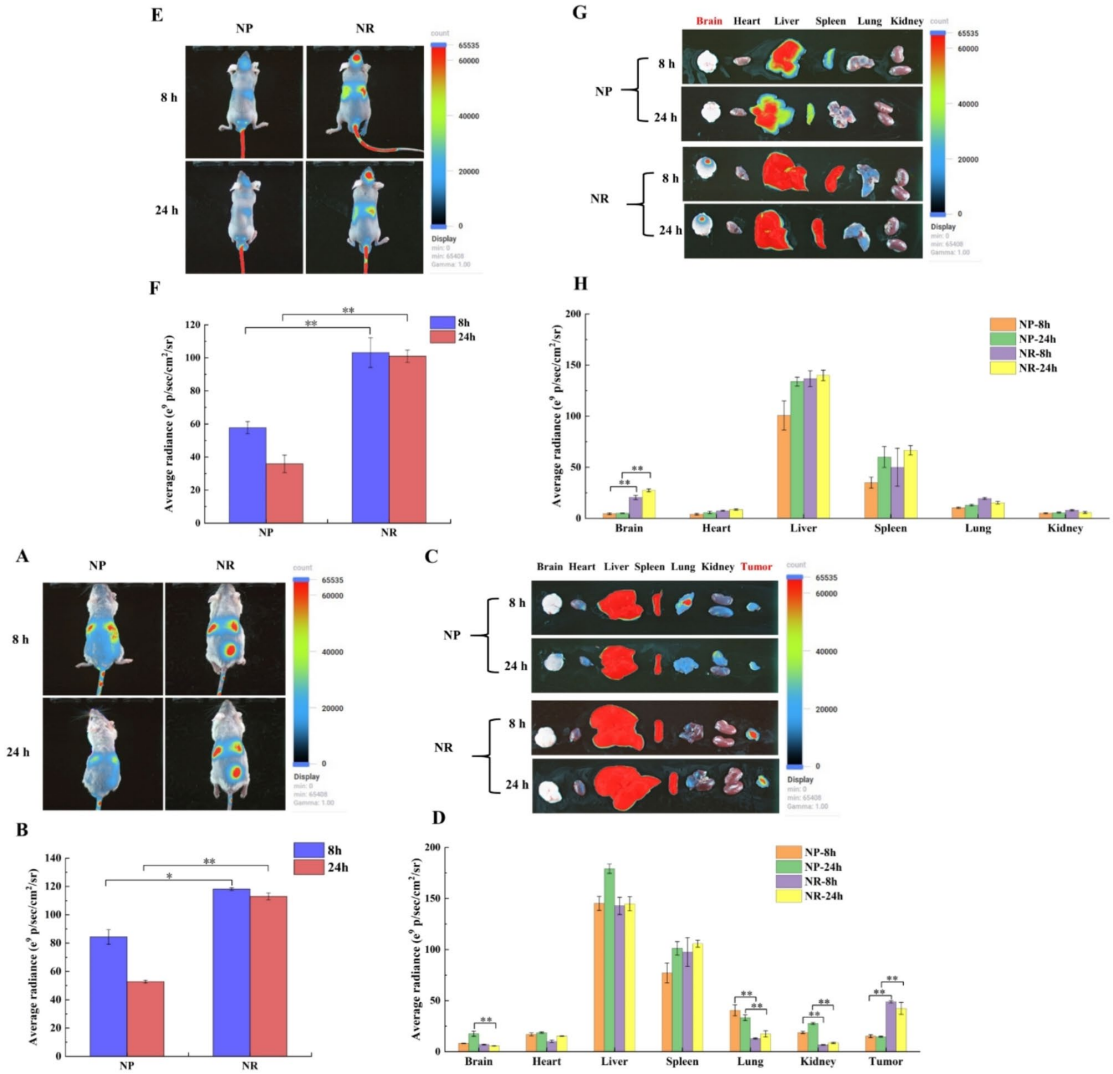
In vivo targeting of engineered nanoplatelets. (**A**) Accumulation of nanoplatelets in subcutaneous glioma model of mice and (**B**) quantitative statistical analysis of tumors; (**C**) Accumulation of nanoplatelets in the organs of mice with subcutaneous tumor and (**D**) quantitative statistical analysis; (**E**) Accumulation of nanoplatelets in orthotopic glioma model of mice and (**F**) quantitative statistical analysis of tumors; (**G**) Accumulation of nanoplatelets in the organs of mice with orthotopic glioma model and (**H**) quantitative statistical analysis; (The results were shown as mean ± standard deviation, *n* = 3; *P < 0.05, **P < 0.01 was statistically significant)

We first constructed a subcutaneous glioma model on Balb/c mice to verify the tumor targeting of NR/TMZ/miR-375 in vivo. NP and NR were labeled with near-infrared dye DiR (λ ex/em: 750/780nm) and injected into mice via caudal vein. In Fig. 7A, strong fluorescence was observed in the tumors of the NR group at 8 h, while the signals of the NP group showed uniform distribution, revealing the strong targeting ability of NR. After 24 h, the fluorescence signals in the NP groups were weakened, but still accumulated in the tumor site of the NR group. In vitro fluorescence imaging and quantitative analysis (Fig. 7B, D) showed strong fluorescence signals in tumors in NR group and the quantitative results implied that the tumor fluorescence intensity increased 3.23 times (8 h) and 2.9 times (24 h) compared with NP group, which further indicated the superior tumor targeting ability of NR. Besides, there was also relatively strong fluorescence in the liver and spleen in both groups, indicating that they were the main metabolic sites of nanoparticles [42, 43].

Subsequently, we constructed a Balb/c orthotopic glioma model and injected DiR-labeled NP and NR through the caudal vein. As depicted in Fig. 7E and G, the NP group exhibited systemic distribution characteristics, whereas NR was mainly accumulated at the brain tumor site, and the fluorescence intensity was 2.79 times higher than that of the NP group at 24 h. Significant brain tumor signal was also detected in the NR group on in vivo imaging, but hardly in the NP group (Fig. 7F, H). At 12 h, fluorescence signals could still be observed in the heart and brain in the NR group, which may produce off-target toxicity. The off-target toxicity could be reduced by targeting ligands modification, increasing the responsiveness of nanoparticles to tumors, and enhancing the stability of nanoparticles. In addition, fluorescent signals could be observed in the tumor sites of NR group mice only 8 h after injection, which was better than 12 h in other studies [24, 44]. The biodistribution results of the two tumor models proved that NR possessed reliable tumor navigation property, which provided theoretical support for the antitumor effect study in vivo.

### In vivo antitumor study of orthotopic glioma

**Fig. 8:**
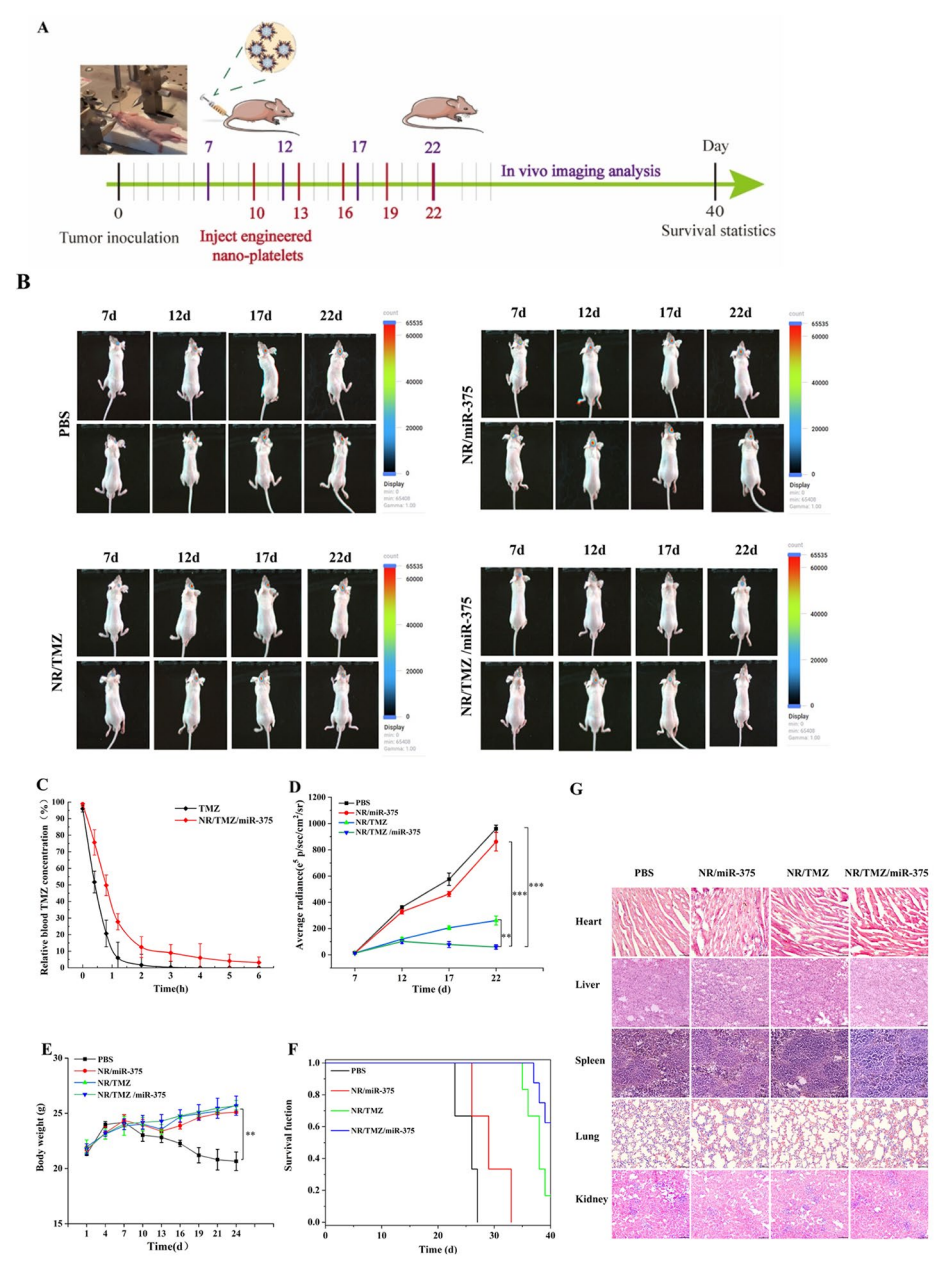
In vivo antitumor study. (**A**) Schematic diagram of the in vivo antitumor study of nanoplatelets. (**B**) Fluorescence imaging of orthotopic gliomas *in vivo.* (**C**) TMZ plasma concentrations after intravenous injection of free TMZ and NR/TMZ/miR-375 in mice. (**D**) Statistical analysis of tumor fluorescence imaging results. (**E**) Statistical results of body weight changes in mice during treatment. (**F**) Survival curve of orthotopic glioma mice. (**G**) Biosafety evaluation of major organs (The results were shown as mean ± standard deviation, *n* = 5; ^**^p < 0.01, ^***^p < 0.001 was statistically significant)

The pharmacokinetics of NR/TMZ/miR-375 were investigated before the antitumor studies (Fig. 8C), the half-life of TMZ was 24 min, while NR/TMZ/miR-375 effectively increased to 56 min, implying NR/TMZ/miR-375 could significantly prolong the drug circulation time in vivo, laying the foundation for in vivo antitumor effect analysis. The orthotopic glioma model was conducted on Balb/c nude mice, and the tumor was monitored through its bioluminescence and imaged by the IVIS system. Mice with tumors were randomly divided into 4 groups with 3 mice in each group, PBS, NR/miR-375, NR/TMZ and NR/TMZ/miR-375 were administered once every 3 days (TMZ: 40 mg/kg, miR-375: 0.2 mg/kg) for total 5 times (Fig. 8A), and the tumors were monitored through fluorescence imaging.

Tumors of mice in different administration groups, as shown in Fig. 8B, revealed that the intensity of fluorescence in each group followed the order: PBS > NR/miR-375 > NR/TMZ > NR/TMZ/miR-375. Quantitative analysis results from Fig. 8D showed that the fluorescence intensity of tumor in NR/TMZ/miR-375 group was relatively stable, while that in other groups increased gradually. And the fluorescence intensity of the NR/TMZ/miR-375 groups decreased 11.7 times compared to the PBS group, highlighting the prominent outcomes of therapy combining gene therapy and chemotherapy.

Figure 8F displays the Kaplan-Meier survival curve, after 40 days of continuous monitoring assay, the median survival time of the PBS group, NR/miR-375 group and NR/TMZ group were 21.5 days, 31 days and 37 days respectively. And 60% of the mice in NR/TMZ/miR-375 group remained alive. Compared with other biomimetic nano systems, such as chemotherapy-delivered exosomes, Wang et al. prepared a biomimetic neutrophil exosome (NEs-Exos) system to deliver doxorubicin (DOX) loaded drugs for glioma treatment [45]. Wu et al. prepared platelet and tumor cell membrane camouflaged β- mangostin loaded NPs (β-PCNPs) for glioma therapy [46]. The median survival time of NEs-Exos and β-PCNPs was 27 days and 33 days, respectively. The combined treatment strategy in this study significantly improved the survival of mice. These results indicated that the nanoplatelets fully exerted the combined treatment effect of miR-375 and TMZ to effectively prolong the survival period of mice. The effectiveness of NR/TMZ/miR-375 in inhibiting glioma growth could be attributed to its efficient targeting ability, successful cross of the blood-brain barrier, and effective penetrate into the glioma. Although NR/TMZ/miR-375 achieved a good antitumor effect, it could not eliminate the tumor completely. Given that the half-life of TMZ loaded in nanoplatelets in this study was about 56 min, it could be increased by surface modification such as coupling PEG and modifying specific ligands to improve the treatment efficiency in future studies. Nanoplatelets could also be combined with immunotherapy such as combination PD-1/PD-L1 antibodies or peptides for better therapeutic effect.

The body weight of the mice and H&E analysis were conducted on the major organs of mice in the administration groups. The body weight of mice in the PBS group gradually decreased over time, whereas mice in the other groups showed no significant fluctuations and even exhibited slight weight gain, indicating that the treatment did not cause significant side effects in the mice (Fig. 8E). The H&E analysis results of major organs were showed in Fig. 8G. No notable differences were observed among the groups, suggesting that the engineered nanoplatelets did not cause significant systemic toxicity to the major organs, thus confirming their favorable biocompatibility.

## Conclusion

In this study, a multifunctional glioma-targeted nanoplatelet combined with chemotherapy and gene therapy was synthesized. Engineered nanoplatelets (NR/TMZ/miR-375) were endowed with glioma targeting ability through the surface modification of RVG, the first-line chemotherapeutic drugs TMZ and therapeutic miR-375 were encapsulated by sonication and electrostatic interaction, respectively. NR/TMZ/miR-375 inherited satisfactory biocompatibility, prolonged circulation time, and good stability of platelets. NR/TMZ/miR-375 was also enable with favorable drug sustained release ability. In vivo antitumor studies of orthotopic glioma revealed that NR/TMZ/miR-375 effectively across the blood-brain barrier and accumulated at the glioma site, and significantly inhibited the proliferation of gliomas, suggesting its bright prospects in glioma treatment.

## Electronic supplementary material

Below is the link to the electronic supplementary material.

Supplementary Material 1

Supplementary Material 2

## Data Availability

No datasets were generated or analysed during the current study.

## References

[1] Madani F, Esnaashari SS, Webster TJ, Khosravani M, Adabi M. Polymeric nanoparticles for drug delivery in glioblastoma: state of the art and future perspectives. J Controlled Release. 2022;349:649–61. 10.1016/j.jconrel.2022.07.023 35878729

[2] Straehla JP, Hajal C, Safford HC, Offeddu GS, Boehnke N, Dacoba TG, et al. A predictive microfluidic model of human glioblastoma to assess trafficking of blood–brain barrier-penetrant nanoparticles. Proc Natl Acad Sci USA. 2022;119:e2118697119. 10.1073/pnas.2118697119 PMC9191661 35648828

[3] Sousa F. Emerging cytokine delivery with nanomedicine for brain cancer treatment. Expert Opin Drug Deliv. 2024;21:513–6. 10.1080/17425247.2024.2347320 38652095

[4] Wang J, Liu Y, Morsch M, Lu Y, Shangguan P, Han L, et al. Brain-targeted aggregation-induced-emission nanoparticles with near-infrared imaging at 1550 nm boosts orthotopic glioblastoma theranostics. Adv Mater. 2022;34:2106082. 10.1002/adma.202106082 34713508

[5] Yin J, Wang X, Ge X, Ding F, Shi Z, Ge Z, et al. Hypoxanthine phosphoribosyl transferase 1 metabolizes temozolomide to activate AMPK for driving chemoresistance of glioblastomas. Nat Commun. 2023;14:5913. 10.1038/s41467-023-41663-2 PMC10516874 37737247

[6] Bausart M, Préat V, Malfanti A. Immunotherapy for glioblastoma: the promise of combination strategies. J Exp Clin Cancer Res. 2022;41:35. 10.1186/s13046-022-02251-2 PMC8787896 35078492

[7] Zou Y, Wang Y, Xu S, Liu Y, Yin J, Lovejoy DB, et al. Brain co-delivery of temozolomide and cisplatin for combinatorial glioblastoma chemotherapy. Adv Mater. 2022;34:2203958. 10.1002/adma.202203958 35738390

[8] Zhang X, Guo Q, Zhao Z, Cheng P, Wu A, Liu H. Engineering prodrug nanoparticles for targeted therapy in heterogeneous glioblastoma. Chem Eng J. 2023;474:145557.

[9] Huang X, Zhu X, Yu Y, Zhu W, Jin L, Zhang X, Li S, Zou P, Xie C, Cui R. Dissecting miRNA signature in colorectal cancer progression and metastasis. Cancer Lett. 2021;501:66–82. 33385486 10.1016/j.canlet.2020.12.025

[10] Wang X, Yang T, Yu Z, Liu T, Jin R, Weng L, Bai Y, Gooding JJ, Zhang Y, Chen X. Intelligent Gold nanoparticles with oncogenic MicroRNA-Dependent activities to manipulate tumorigenic environments for synergistic tumor therapy. Adv Mater. 2022;34:2110219. 10.1002/adma.202110219 35170096

[11] Zheng T, Wang W, Mohammadniaei M, Ashley J, Zhang M, Zhou N, Shen J, Sun Y. Anti-MicroRNA-21 oligonucleotide loaded spermine-modified Acetalated Dextran nanoparticles for B1 receptor-targeted gene therapy and antiangiogenesis therapy. Adv Sci. 2022;9:2103812. 10.1002/advs.202103812 PMC8844571 34936240

[12] Xu B, Mei J, Ji W, Huo Z, Bian Z, Jiao J, Li X, Sun J, Shao J. MicroRNAs involved in the EGFR pathway in glioblastoma. Biomed Pharmacother. 2021;134:111115. 33341046 10.1016/j.biopha.2020.111115

[13] Yang T, Zhao P, Rong Z, Li B, Xue H, You J, et al. Anti-tumor efficiency of lipid-coated cisplatin nanoparticles co-loaded with MicroRNA-375. Theranostics. 2016;6:142–54. 10.7150/thno.13130 PMC4679361 26722380

[14] Zhu Y, Liang G, Sun B, Tian T, Hu F, Xiao Z. A novel type of self-assembled nanoparticles as targeted gene carriers: an application for plasmid DNA and antimicroRNA oligonucleotide delivery. Int J Nanomed. 2016;11:399–411. 10.2147/IJN.S84927 PMC4734819 26869785

[15] Fan X, Shi L, Yang Z, Li Y, Zhang C, Bai B, et al. Targeted repair of spinal cord injury based on miRNA-124‐3p–loaded mesoporous silica camouflaged by stem cell membrane modified with rabies virus glycoprotein. Adv Sci. 2024;11:2309305. 10.1002/advs.202309305 PMC11151008 38509833

[16] Jahan S, Karim ME, Chowdhury EH. Nanoparticles targeting receptors on breast cancer for efficient delivery of chemotherapeutics. Biomedicines. 2021;9:114. 33530291 10.3390/biomedicines9020114 PMC7910939

[17] Karim ME, Tha KK, Othman I, Borhan Uddin M, Chowdhury EH. Therapeutic potency of nanoformulations of siRNAs and shRNAs in animal models of cancers. Pharmaceutics. 2018;10:65. 29861465 10.3390/pharmaceutics10020065 PMC6026921

[18] Wang X, Liang GF, Hao XQ, Feng SY, Dai L, An JL, et al. Bioinspired drug delivery carrier for enhanced tumor-targeting in melanoma mice model. J Biomed Nanotechnol. 2019;15:1482–91. 31196352 10.1166/jbn.2019.2786

[19] Wang Y, Li W, Li Z, Mo F, Chen Y, Iida M, Wheeler DL, Hu Q. Active recruitment of anti–PD-1–conjugated platelets through tumor-selective thrombosis for enhanced anticancer immunotherapy. Sci Adv. 2023;9:eadf6854. 36989364 10.1126/sciadv.adf6854 PMC10058243

[20] Liang G, Kan S, Zhu Y, Feng S, Feng W, Gao S. Engineered exosome-mediated delivery of functionally active miR-26a and its enhanced suppression effect in HepG2 cells. Int J Nanomed. 2018;13:585–99. 10.2147/IJN.S154458 PMC5796471 29430178

[21] Migliavacca M, Correa-Paz C, Pérez-Mato M, Bielawski P-B, Zhang I, Marie P, et al. Thrombolytic therapy based on lyophilized platelet-derived nanocarriers for ischemic stroke. J Nanobiotechnol. 2024;22:10. 10.1186/s12951-023-02206-5 PMC10763438 38166940

[22] Luo L, Zhang B, Tao F, Chen Z, Ye Q, Zhao X, et al. Perfluorotributylamine-loaded albumin nanoparticles downregulate platelet-derived TGFbeta to inhibit tumor metastasis. ACS Nano. 2023;17:15388–400. 37526429 10.1021/acsnano.3c00295

[23] Xu H-Z, Li T-F, Ma Y, Li K, Zhang Q, Xu Y-H, Zhang Y-C, Zhao L, Chen X. Targeted photodynamic therapy of Glioblastoma mediated by platelets with photo-controlled release property. Biomaterials. 2022;290:121833. 36201945 10.1016/j.biomaterials.2022.121833

[24] Jiang Z, Zhang H, Zhang W, Zhang Y, Cui Y, Mei L, Wang Q. Smart platelet-based biohybrid delivery system for magnetic-guided targeted delivery and enhanced photothermal-chemo therapy against glioma. Nano Today. 2024;56:102295.

[25] Yin N, Wang Y, Huang Y, Cao Y, Jin L, Liu J, et al. Modulating nanozyme-based nanomachines via microenvironmental feedback for differential photothermal therapy of orthotopic gliomas. Adv Sci. 2023;10:2204937. 10.1002/advs.202204937 PMC9875674 36437111

[26] Mariappan A, Goranci-Buzhala G, Ricci-Vitiani L, Pallini R, Gopalakrishnan J. Trends and challenges in modeling glioma using 3D human brain organoids. Cell Death Differ. 2021;28:15–23. 33262470 10.1038/s41418-020-00679-7 PMC7707134

[27] Liang G, Li G, Wang Y, Lei W, Xiao Z. Aberrant miRNA expression response to UV irradiation in human liver cancer cells. Mol Med Rep. 2014;9:904–10. 24431000 10.3892/mmr.2014.1901

[28] Fei XF, Zhang QB, Dong J, Diao Y, Wang ZM, Li RJ, Wu ZC, Wang AD, Lan Q, Zhang SM, Huang Q. Development of clinically relevant orthotopic xenograft mouse model of metastatic lung cancer and glioblastoma through surgical tumor tissues injection with trocar. J Experimental &clinical cancer Res. 2010;29:84. 10.1186/1756-9966-29-84 PMC2907866 20587035

[29] Qi L, Baxter P, Kogiso M, Zhang H, Braun FK, Lindsay H, et al. Direct implantation of patient brain tumor cells into matching locations in mouse brains for patient-derived orthotopic xenograft model development. Cancers. 2024;16:1716. 10.3390/cancers16091716 PMC11083000 38730671

[30] Lai TH, Wenzel B, Dukic-Stefanovic S, Teodoro R, Arnaud L, Maisonial-Besset A, et al. Radiosynthesis and biological evaluation of [(18)F]AG-120 for PET imaging of the mutant isocitrate dehydrogenase 1 in glioma. Eur J Nucl Med Mol Imaging. 2024;51:1085–96. 10.1007/s00259-023-06515-7 PMC10881675 37982850

[31] Zhu Q, Liang P, Meng H, Li F, Miao W, Chu C, et al. Stabilization of pin1 by USP34 promotes Ubc9 isomerization and protein sumoylation in glioma stem cells. Nat Commun. 2024;15:40. 38167292 10.1038/s41467-023-44349-x PMC10762127

[32] Shi Y, Xu X, Yu H, Lin Z, Zuo H, Wu Y. Defined positive charge patterns created on DNA nanostructures determine cellular uptake efficiency. Nano Lett. 2022;22:5330–8. 35729707 10.1021/acs.nanolett.2c01316

[33] Zhang M, Chen X, Li C, Shen X. Charge-reversal nanocarriers: an emerging paradigm for smart cancer nanomedicine. J Controlled Release. 2020;319:46–62. 10.1016/j.jconrel.2019.12.024 31846619

[34] Xu J, Yang X, Ji J, Gao Y, Qiu N, Xi Y, et al. RVG-functionalized reduction sensitive micelles for the effective accumulation of doxorubicin in brain. J Nanobiotechnol. 2021;19:251. 10.1186/s12951-021-00997-z PMC8379803 34419071

[35] Su W, Zhu S, Chen K, Yang H, Tian M, Fu Q, et al. Overexpressed WDR3 induces the activation of hippo pathway by interacting with GATA4 in pancreatic cancer. J Exp Clin Cancer Res. 2021;40:88. 10.1186/s13046-021-01879-w PMC7923337 33648545

[36] Gan J, Liu S, Zhang Y, He L, Bai L, Liao R, et al. MicroRNA-375 is a therapeutic target for castration-resistant prostate cancer through the PTPN4/STAT3 axis. Exp Mol Med. 2022;54:1290–305. 36042375 10.1038/s12276-022-00837-6 PMC9440249

[37] Ding P, Liang B, Shou J, Wang X. lncRNA KCNQ1OT1 promotes proliferation and invasion of glioma cells by targeting the miR‑375/YAP pathway. Int J Mol Med. 2020;46:1983–92. 33125099 10.3892/ijmm.2020.4760 PMC7595660

[38] Ren Z, Li J, Zhao S, Qiao Q, Li R. Knockdown of MCM8 functions as a strategy to inhibit the development and progression of osteosarcoma through regulating CTGF. Cell Death Dis. 2021;12:376. 33828075 10.1038/s41419-021-03621-y PMC8027380

[39] Choi KM, Kim B, Lee SM, Han J, Bae HS, Han SB, et al. Characterization of gastric cancer-stimulated signaling pathways and function of CTGF in cancer-associated fibroblasts. Cell Commun Signaling. 2024;22:8. 10.1186/s12964-023-01396-7 PMC10763493 38167009

[40] Zhang Y, Wang Y, Ji H, Ding J, Wang K. The interplay between noncoding RNA and YAP/TAZ signaling in cancers: molecular functions and mechanisms. J Experimental Clin Cancer Res. 2022;41:202. 10.1186/s13046-022-02403-4 PMC9199231 35701841

[41] Qiao S, Cheng Y, Liu M, Ji Q, Zhang B, Mei Q, et al. Chemoattractants driven and microglia based biomimetic nanoparticle treating TMZ-resistant glioblastoma multiforme. J Controlled Release. 2021;336:54–70. 34129862 10.1016/j.jconrel.2021.06.015

[42] Kumar M, Kulkarni P, Liu S, Chemuturi N, Shah DK. Nanoparticle biodistribution coefficients: a quantitative approach for understanding the tissue distribution of nanoparticles. Adv Drug Delivery Rev. 2023;194:114708. 36682420 10.1016/j.addr.2023.114708

[43] Hu D, Yang RY, Wang GD, Li H, Fan XL, Liang GF. Emerging strategies to overcome current CAR-T therapy dilemmas - exosomes derived from CAR-T cells. Int J Nanomed. 2024;19:2773–91. 10.2147/IJN.S445101 PMC10959326 38525009

[44] Qiao S, Cheng Y, Liu M, Ji Q, Zhang B, Mei Q, Liu D, Zhou S. Chemoattractants driven and microglia based biomimetic nanoparticle treating TMZ-resistant glioblastoma multiforme. J Controlled Release. 2021;336:54–70. 10.1016/j.jconrel.2021.06.015 34129862

[45] Wang J, Tang W, Yang M, Yin Y, Li H, Hu F, et al. Inflammatory tumor microenvironment responsive neutrophil exosomes-based drug delivery system for targeted glioma therapy. Biomaterials. 2021;273:120784. 10.1016/j.biomaterials.2021.120784 33848731

[46] Wu L, Li Q, Deng J, Shen J, Xu W, Yang W, et al. Platelet-tumor cell hybrid membrane-camouflaged nanoparticles for enhancing therapy efficacy in glioma. Int J Nanomed. 2021;16:8433–46. 10.2147/IJN.S333279 PMC8727453 35002237

