# Supplementary material for: Nanoplatelets modified with RVG for targeted delivery of miR-375 and temozolomide to enhance gliomas therapy
Source: J Nanobiotechnology. 2024 Oct 15;22:623. doi: 10.1186/s12951-024-02895-6 (PMC11476726; doi:10.1186/s12951-024-02895-6)
Supplement: Supplementary file 1 — Supplementary Material 1 [file 12951_2024_2895_MOESM1_ESM.docx]

## Supplement Information:

Table S1:
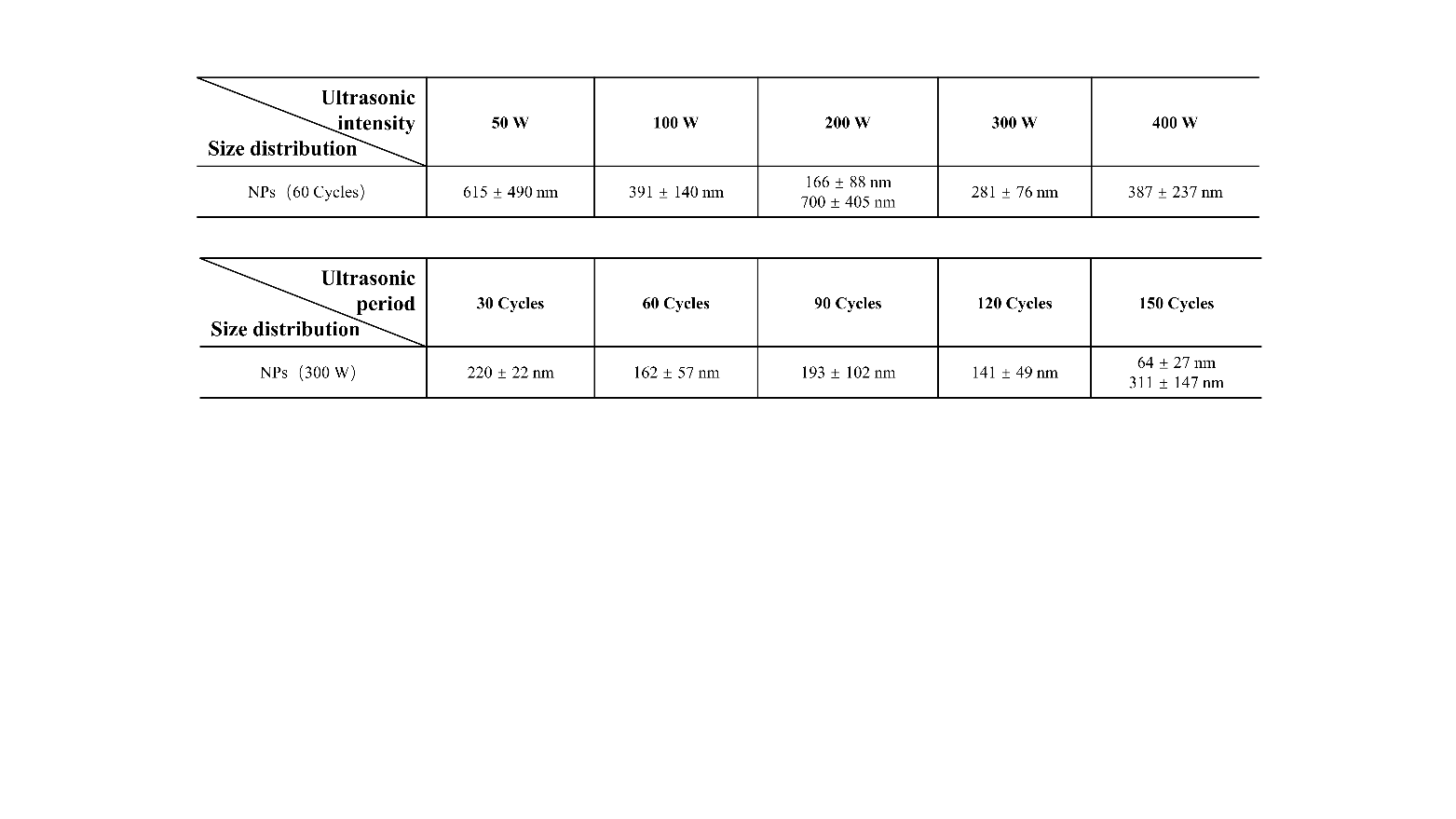
 Particle size distribution of NPs under different ultrasonic conditions.

Figure S2:


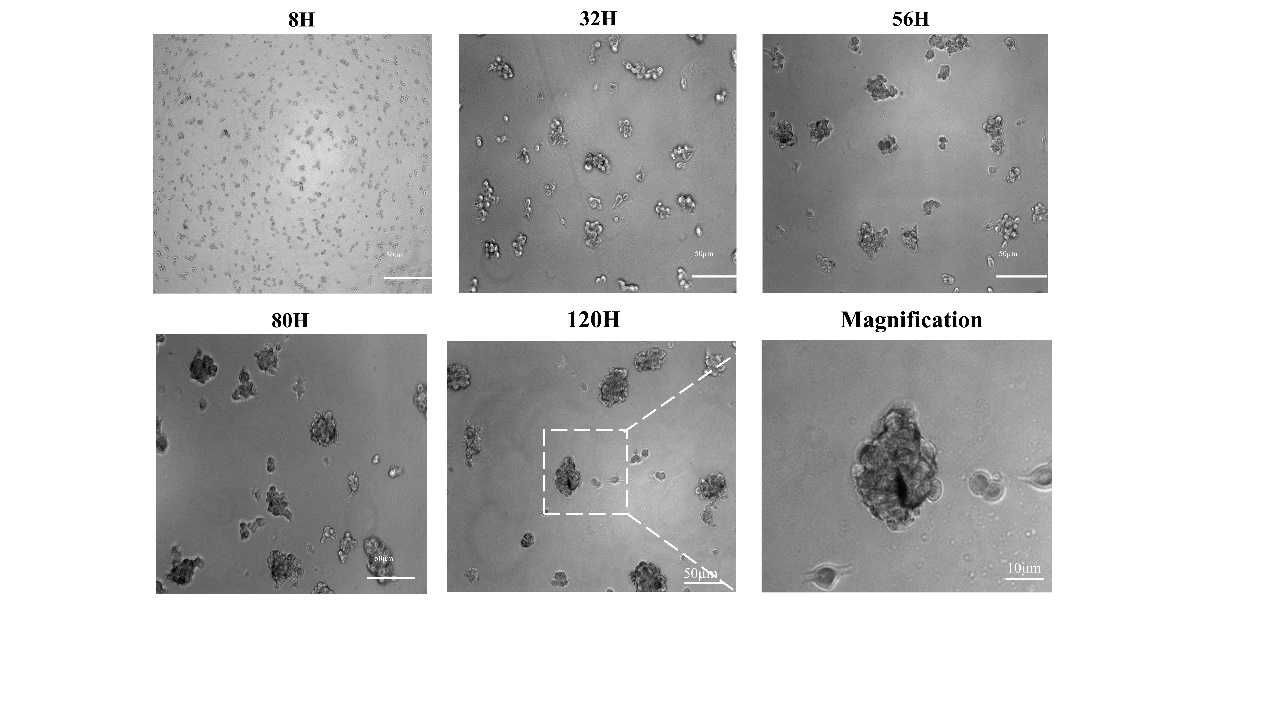


Figure S2 Microscopically photographed U87 cell spheres.


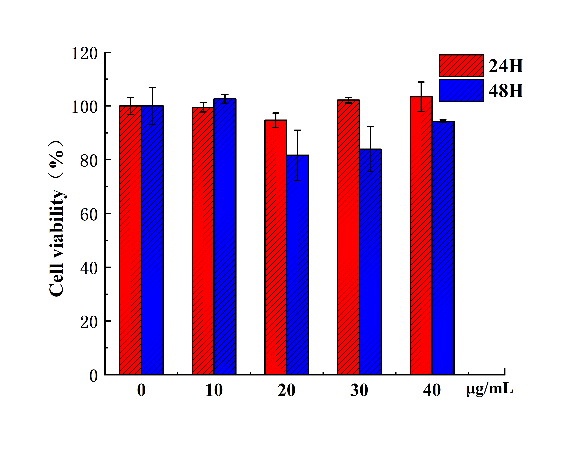
Figure S3:

Figure S3 Effects of different concentrations of nanoplatelets on cell activity.
